# Impact of stakeholder pressure on digital process innovation: An empirical analysis

**DOI:** 10.1371/journal.pone.0307528

**Published:** 2024-07-23

**Authors:** Yi Jin, Xun Yao, Minying Huang

**Affiliations:** Business School, Southwest Minzu University, Chengdu, China; SEGi University Kota Damansara, MALAYSIA

## Abstract

Digital technologies can bring about fundamental changes in corporate processes, which may result in a shift from process innovation to digital process innovation. However, owing to resource constraints and various stakeholders, digital process implementation is extremely challenging for firms. Based on stakeholder theory, this study explores whether and how stakeholder pressure for digitalization can facilitate corporate digital process innovation and unravels the mediating effect of routine reconfiguration and the moderating effect of strategic flexibility. The findings from a survey of 351 firms prove that stakeholder pressure for digitalization can facilitate corporate digital process innovation via routine reconfiguration. Moreover, this study finds that increased strategic flexibility can strengthen the positive mediating effect of routine reconfiguration. The findings contribute to the deep understanding of digital process innovation and offer a boundary condition for the effectiveness of stakeholder pressure.

## Introduction

Digital technologies are disrupting industries across the globe and exerting a massive impact on the value chain and stakeholders’ actions [1–3]. For example, the Chinese government implemented several policies, such as the *Development Plan for the New Generation of Artificial Intelligence*, to promote the application of artificial intelligence (AI). Meanwhile, leading manufacturing firms are motivating their first- and lower-tier suppliers to engage in digital technology adoption. However, though firms may gain potential benefits from digital transformation, digitalization is challenging. Thus, unpacking the factors that can drive digitalization would be timely and worthwhile.

Digital process innovation, as an extension of traditional process innovation, has become crucial for firms that want to stay or be on top of their game [4–6]. Digital process innovation, which is also known as digitally-enabled process innovation, refers to the use of different digital technologies (e.g., automation, the Internet of Things, cloud computing, AI) and solutions in a firm’s business process [2, 7], which suggests the combined effects of the firm’s ability and implementation to adapt to the changed context. Successful digital process innovation can increase productivity, enhance efficiency, improve security, and drive the successful development of other forms of digital innovation [2]. Despite the importance of digital process innovation, prior research paid considerable attention only to antecedents of digital innovation, such as digital green network embedding, digital platforms, and government initiatives [8, 9]. Such fruitful findings laid a solid foundation for understanding digital process innovation. However, prior studies also suggested that digital process innovation differs from digital innovation, including their targeted goals, strategic foci, and value-creation approaches [10]. Moreover, [7] claimed that process innovation has received less conceptual and empirical attention than product innovation and thus called for attention to be paid to digital process innovation. Hence, insights into digital process innovation must be gleaned from new observations and ideas.

As digitalization becomes prevalent, firms are being pressured by stakeholders to invest in digitalization to cope with the changes sweeping the value chain. Stakeholders are generally defined as “any group or individual who can affect or be affected by the achievement of the organizational objectives” [11]. Stakeholder pressure can exert influence and control a firm based on its power, legitimacy, and critical resources [12]. Previous studies described stakeholder pressure as a firm’s perception and interpretation of the significance of stakeholders’ concerns about environmental protection [13]. Previous studies focused on service research and green issues and found that stakeholder pressure can influence a firm’s supply chain policies and general practices, environmental practices, knowledge management activities, organizational learning, demand responsiveness, and performance [13, 14]. Owing to the importance of dealing effectively with the interests of stakeholders [15], many firms feel pressure from stakeholders’ demand to go digital [12, 16]. Thus, this study conceptualizes stakeholder pressure as the influence of stakeholders’ concerns about digitalization on a firm’s decisions. Based on the degree of relevance to a firm’s operation, prior stakeholder studies focused on customers, suppliers, employees, the government, shareholders, creditors, or competitors [15]. However, few studies linked stakeholder pressure with the digital process innovation of a firm and opened the black box. The lack of digitalization studies can impede progress in understanding firms’ digital investment and implementation.

To answer the call to adopt a stakeholder approach to analyze a firm digitalization [16], we propose a theoretical model that integrates stakeholder pressure and digital process innovation and explores the mediating effect of routine reconfiguration and the moderating effect of strategic flexibility on the above relationship. Stakeholders exert pressure on a firm to increase its digital process innovation to adapt to supply chain sustainability and increase competitiveness [17]. The instrumental perspective of stakeholder theory expects firms to consider the concerns of stakeholders as opportunities to increase competitive advantage and respond to such concerns by engaging in behavior that can address their demands [18, 19]. Thus, we investigated how different levels of digitalization pressure from stakeholders may impact a firm’s digital process innovation. Stakeholder pressure will not guarantee successful innovation. Firms must create new workflows, procedures, and regular activities in their operation, that is, routine reconfiguration, to meet the requirements of digital process innovation. Furthermore, firms need a flexible strategy (i.e., strategic flexibility) to ensure their rapid switch to a new project and ability to offer adequate resources when they decide to invest in digital process technologies. Thus, we shift the focus from process innovation and digital innovation to digital process innovation, which was largely overlooked in digitalization studies, and advance the nuanced understanding of digital process innovation.

To examine the theoretical model, we employed a widely used empirical method (i.e., a questionnaire) for addressing innovation management issues to acquire and analyze data. We obtain 351 valid samples, examine the reliability and validity of all the variables to guarantee the effectiveness of the data, and finally perform regression analysis. The results show that the theoretical model is valid. First, the findings emphasize the role of stakeholder pressure in affecting digital process innovation, which is in line with those of previous stakeholder and digital innovation studies [4, 9, 20]. Second, the results prove the mediating effect of routine reconfiguration. The finding can improve our understanding of the relationship between stakeholder pressure and digital process innovation, because it reveals that routine reconfiguration is a firm’s feedback and response to pressure from stakeholders, and such organizational actions can transfer pressure from stakeholders to digital applications and digital process innovation performance. This finding partially agrees with that of [20]. Third, our study contributes to instrumental stakeholder theory by clarifying that response to stakeholders’ demands and concerns does not guarantee an opportunity to create and the successful transformation of certain organizational outcomes. Instead, it relies on a firm’s strategic flexibility to switch strategies and redeploy resources. Different from previous studies, which typically regarded strategic flexibility as a mediator [21, 22], our study find that the strategic flexibility of a firm plays a boundary role. That is, a firm with high strategic flexibility can effectively redeploy organizational resources to transfer pressure from stakeholders to digital behaviors and thus increase its digital process innovation.

The rest of this paper organized as follows: The literature review and hypothesis development section reviews the relevant literature and proposes the research hypotheses, and research and data methodology section describes the research design, sample collection process, measurements, and data processing strategies. The data analysis section analyze the regression results and tests the research hypotheses, and conclusion and implications section summarizes and discusses the implications of the empirical results.

## Literature review and hypothesis development

### From process innovation to digital process innovation

Process innovation is generally defined as the implementation of a new or significantly improved production or delivery method. According to the prediction [23], the development of a dominant design will change the focus of competition from product innovation to price/cost reduction involving process innovation [24]. Following this logic, the goals of process innovation are primarily efficiency-driven [25], including increasing the quality of products, reducing expenses, and strengthening operational resilience [2, 26]. Thus, process innovation has been recognized as a crucial strategy for firms to enhance their new product performance [27], green supply chain performances [28], financial performance [29], productivity, and competitiveness [30]. Regarding the enablers of process innovation, previous studies proved that technological factors (e.g., technological capability), knowledge factors (e.g., knowledge search and suppliers’ knowledge), market factors, organizational factors (e.g., firm size and family involvement in management), inter-organizational factors (e.g., collaborative innovation networks), industrial factors (e.g., dominant design), and institutional factors (e.g., government intervention) are associated with increased process innovation [24, 26, 27, 31, 32].

In this digital era, process innovation has been extended to digital process innovation, which is also known as digitally enabled process innovation. According to [2, 7], digital process innovation refers to the implementation of digital technologies, such as Industry 4.0, AI, big data, 3D printing, autonomous solutions, and analytics, to enable new or significantly improved business processes, such as production or delivery methods. Digital technologies may digitalize non-digital processes (e.g., the geographic information system enabled the process of finding, reserving, and paying for a ride) and digital processes (e.g., the blockchain system changed the transfer of money on a conventional digital network) [33].

According to [34], digital process innovation has three features. First, digital process innovation blurs spatial and temporal boundaries. For example, 3D technology allows different actors to participate in the business process of innovation at different times and locations [35]. Second, digital process innovation blurs the boundaries of digital product innovation. Product and process innovation in the digital era is intrinsically interdependent [2]. Digital product innovation is an academic focus [36]; thus, in this study, we identify and explore only the development of digital process innovation. Third, the reprogrammability of digital technologies may result in many derivative innovations in digital process innovation.

Despite the potential benefits of digital process innovation, it has received little conceptual and empirical attention. Several studies focused on digital process innovation in process industries (e.g., the mining industry and the steel industry) and found that ecosystem strategies, infrastructure, methodological definitions, preparation for predictive and analytical readiness, proactive management practices, and planning for the digital maturity of each function and department are enablers of digital process innovation [2, 7]. Other studies suggested that the openness of a firm’s specialized search can positively affect its digital process innovation via its absorptive capacity [4]. Hence, research focused on the antecedents of digital process innovation in other industries should be increased to deepen the nuanced understanding of this issue.

### Stakeholder pressure

According to stakeholder theory, a firm is a nexus of relationships among its primary stakeholders [18]. Primary stakeholders, which can influence organizational goals to create value, include supply chain partners (suppliers and customers), competitors, shareholders, the government, creditors, and employees [37]. Such stakeholders exert influence by offering important resources or power and legitimacy or both [12]. Therefore, stakeholders’ expectations, demands, and requirements may lead to pressure that corporations should manage carefully. In this digital era, primary stakeholders have become increasingly empowered to implicitly or explicitly exert pressure on organizations to call for digitalization. For example, [17] posited that firms must adapt to the disruptions caused by digitalization to ensure their long-term survival, which can result in competitive pressure. Thus, our study focuses on stakeholder pressure for digitalization.

Previous stakeholder studies posited that stakeholder theory can be divided based on firms’ motives for considering the role of stakeholders [38]. For economic returns, instrumental stakeholder theory suggests that addressing the interests of various stakeholders will enable firms to achieve their financial performance goals through improved reputation and increased trust from stakeholders [39, 40]. Meanwhile, moral stakeholder theory regards ethics as “the right thing to do” [38]. One possible explanation for the differences is that previous stakeholder studies are linked closely with corporate social responsibility and the adoption of environmental practices, for which the motives for corporate behavior matter. However, in other contexts, moral or financial reasons are not contradictory but reciprocal and mutually supportive [15]. Therefore, in our study, we consider the importance of stakeholders as a synthesis of various factors in a broad manner.

Previous studies highlighted the predictive role of stakeholder pressure in explaining innovation issues, and thus, laid a solid foundation for the relationship between stakeholder pressure for digitalization and innovation outcomes. For example, according to [41], stakeholder pressure is related to the innovative capacity of SMEs. Stakeholder pressure also can positively influence green innovation by improving green dynamic capabilities [42] and lead to the implementation of certain management systems through the influence of coercive pressure and rewards motivation from external stakeholders, which then affect green innovation [43]. In essence, stakeholder pressure can induce organizational behaviors that may further affect innovation decisions and outcomes.

### Stakeholder pressure for digitalization and digital process innovation

In the specific context of a firm’s digital process innovation, stakeholders can make a wide range of requests to firms. In many countries, the government, as a strong stakeholder, pushes firms to establish digital infrastructure and adopt digital technologies for their manufacturing process. For example, in 2015, the Chinese government issued the *Notice on the Action Outline for Promoting the Development of Big Data*, to develop industrial big data and promote its application in all aspects of the product life cycle (from industrial R&D design, production, and manufacturing, operation management, and marketing to after-sales service) and industry chain. Thus, the Chinese government not only set up pilot firms, provided project subsidies, and set role models (i.e. selected excellent application cases), but also built solid digital infrastructures, which can spur firms’ digital process innovation [44].

Customers and suppliers are also strong stakeholders in the digital ecosystem to which they belong [2]. Customers’ pressure can create unique opportunities for firms to enhance their digital processes through customer engagement, information sharing, customization, and product personalization [45]. For example, Foxconn which is a lighthouse factory and the first to utilize multiple digital technologies in the industry to form a digitalized factory, exerts a profound impact on thousands of cooperative upstream and downstream firms in its supply chain. To achieve real-time communication of production demand information and reduce information flow costs, Foxconn requires its suppliers to use the same system for quality management, procurement, logistics, and other information. Foxconn engages in its suppliers’ digital process, shares necessary information with its suppliers, and provides necessary support (e.g., by holding intelligent manufacturing acceleration camps), which forced suppliers to digitalize and improve their business process, thereby, spurring their digital process innovation.

Employees generally ask for standard work hours, a safe working environment, and a flexible job design, which can encourage firms to adopt automation and intelligent manufacturing technologies to innovate, then realize agile production. As [17] suggested, pressure from a competitor can exert a considerable impact on a firm’s digital process innovation efforts, because employees will be afraid to fail and be replaced. Besides, because firms, especially digital entrepreneurship, rely on external financing sources to foster their growth [46], pressure from financiers matters. Firms must develop new business processes and demonstrate their capacity to operate in the present while paying attention to the future [47]. As a result, firms pursuing support from venture capital will be likely to modify and improve their existing business processes by using digital technologies.

Pressure on firms comes from stakeholders to use digital technologies for R&D can modify their existing production and delivery processes, and create new process patterns. Such pressure can shape a firm’s managerial digital attention through initiative-induced opportunities or resource accumulation [8]. Moreover, to a certain degree, stakeholder pressure can convey long-term vision, valuable information, specialized knowledge, and digital process innovation application scenarios [17], which may inspire engagement in digital process innovation. Therefore, stakeholder pressure for digitalization will enable the digital process innovation of a firm. Based on the above discussion, we propose the following hypothesis:

H1. *A positive relationship exists between stakeholder pressure for digitalization and a firm’s digital process innovation*.

### Mediating role of routine reconfiguration

As a repetitive, recognizable pattern of the interdependent actions of an organization [48], organizational routines imply organizational memories, knowledge, and resources [49], whereas routine reconfiguration “involves the retention, modification, deletion, and addition of actions that compose an organizational routine” [50]. Such reconfiguration suggests that a “creative project” trajectory is being followed to accomplish new tasks or create new routines by envisioning a new end state and attaining objectives through emergent action [49, 51].

Numerous examples show that ignoring the demands of customers, suppliers, investors, and the government can lead to failure, which can increase pressure on a firm. Such pressure will not be alleviated unless the firm responds to it through appropriate actions. Inevitably, stakeholder pressure for digitalization will result in the routine reconfiguration of a firm. First, as a relatively stable pattern, a routine requires either internal or external stimuli to induce changes [52], whereas pressure from stakeholders serves as a stimulus to modify existing routines. Pressure from stakeholders can prompt corporations to introduce computer-aided software, new equipment, standard operating procedures, work instructions and workflow, and the corresponding human resources into their operation. Thus, the skills, experience, domain knowledge, and management styles embedded in the old business will not be applicable to the new context, and routine reconfiguration will be necessary.

Second, though a routine exhibits stability, pressure from stakeholders will provide signals, information, resources, and space for routine variation. For example, the expectations and information released by the government in formal and informal situations can encourage firms to transform their maintenance practices and work behaviors and envision novel practices and experiences. Similarly, because of the high level of interdependence between supply chain firms and the stakeholders [53], their meetings can be a venue for shaping emerging standards by addressing the apparent objectivity of a “matter of concern” and translating it into a “matter of fact.” Therefore, pressure from stakeholders can encourage corporations to intersect and reconfigure existing routines in response to potential failure and costs. Based on the above discussion, we propose the following hypothesis:

H2. *A positive relationship exists between stakeholder pressure for digitalization and a firm’s routine reconfiguration*.

As a key enabler of digital process innovation, stakeholder pressure for digitalization can have a significant impact on the input of information, resources, and knowledge inputs required for digital process innovation, as well as on innovation decisions. However, the digital innovation of organizations does not occur in a vacuum [54], instead, it builds on the digital infrastructure, and the adoption of digital technologies for innovation, which can lead to change in and the reconfiguration of routines. The introduction of digital technologies into various business processes will lead to the reconfiguration of various complex and implicit organizational routines. Routine configuration is a process through which organizations eliminate outdated and inefficient practices after an external search and selection; disrupt existing internal consensus, norms, and behaviors; and form new routines that match existing business processes [51]. A routine trajectory is based on past repetition and a stable pattern of action to keep work on track [49]; thus, routine configuration is typically a reactive choice driven by external forces. Stakeholders’ demand and the necessary response can drive organizational routines restructuring, because stakeholders’ demands for digitalization can help firms determine their digital transformation direction and obtain information and resources related to business processes. The dissemination, analysis, and use of information within an organization will shake the foundations of existing organizational routines while reducing the trial-and-error costs of the digital transformation of business processes, and promoting digital process innovation. Based on the above discussion, we propose the following hypothesis:

H3. *Routine reconfiguration will mediate the relationship between stakeholder pressure and a firm’s digital process innovation*.

### Moderating role of strategic flexibility

Strategic flexibility refers to a set of capabilities of a corporation to adjust its strategic decisions in proactive or reactive response to new opportunities, threats, and changes in the internal and external environment [21, 55]. Strategic flexibility generally involves capabilities for identifying major changes, reallocating and reconfiguring existing resources and processes, and quickly committing resources and processes to changes, thereby resulting in a wide array of strategic options [56]. Thus, firms that demonstrate strategic flexibility have flexible resource pools that are available in other contexts and less switching time and costs, which was suggested by [57] as resource flexibility. Moreover, strategic flexibility can equip firms with the ability to coordinate alternative uses of flexible resources [55, 58].

According to dynamic capability theory, in responding to stakeholders’ demands for digitalization, a corporation should renew, augment, and adapt to the new environment by controlling and exploiting its resources. Thus, a firm’s existing resources play an important role in reconfiguring new routines. When strategic flexibility is low, it will not be able to use existing resources to create new routines that are applicable to a digital business environment. High asset specialization and resource rigidity can inhibit a firm from promptly halting or reversing resource commitments [59] and thus constrain its response to stakeholders’ digital demands and its establishment of routines, procedures, and rules related to its digital operation. As a firm’s strategic flexibility increases, its existing resources will become adequately flexible to be effectively and easily recombined for new purposes [21], which can help the firm quickly adapt its routines over time and obtain a first-mover advantage [60]. Thus, flexible resources will likely be valuable to and have strong implications for firms when responding to stakeholders’ demands.

In addition, the support provided by strategic flexibility will give rise to the variation and retention involved in the creation and intentional reconfiguration of routines. As its strategic flexibility increases, a firm will be able to integrate and deploy internal and external resources efficiently, identify and hold business opportunities, and decrease the time and resources spent on searching for digital resources and resistance to the cognitive screening mechanism requirements, which are critical to the successful reconfiguration of routines. Its strategic flexibility can provide a firm with adequate resources and capabilities to explore, experiment, entrench, and enact. Thus, a firm with high strategic flexibility can exploit flexible resources inside and outside the firm to reconfigure its routines and further advance its digital process innovation. Therefore, we propose the following hypotheses:

H4a. *A firm’s strategic flexibility will positively moderate the relationship between stakeholder pressure and the firm’s routine reconfiguration*.
H4b. *A firm’s strategic flexibility will positively moderate the mediating effect of routine reconfiguration on the relationship between stakeholder pressure and the firm’s digital process innovation*.

**Fig 1:**
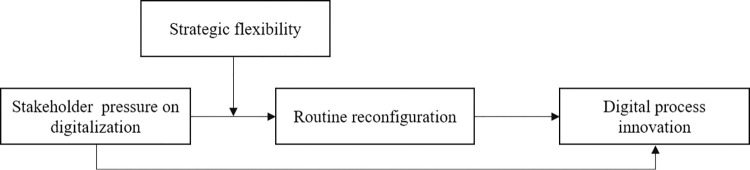
The conceptual model of this paper.

The overall conceptual model can be seen in Fig 1.

## Research and data methodology

### Sample and data collection

We conducted a survey investigation on 351 manufacturing and service firms in China. Manufacturing firms are considered to be a potential sector for digital innovation because digitalization has prospered in factory networks and integrated value chains [61, 62]. Moreover, digitalization will allow firms to transition to digital servitization, which may prompt the creation of novel business processes [3]. Thus, we acknowledge the vast digital process innovation potential of the service sector.

To examine the hypotheses, we selected mature questionnaires from previous studies. Considering semantic loss in translation, we employed the back-translation method to ensure the quality of the questionnaire. Next, we cooperated with two experts in the manufacturing industry and management issues to modify the wording of the questionnaire. To collect the data, our research team visited three digitalization summits and two industry fairs in Southwest and Southeast China, which attracted thousands of firms. With the help of instant messaging platforms, we established and maintained contact with the managers or owners of different manufacturing companies before and after the summits. We sent the e-survey to the companies that we approached directly. Meanwhile, we randomly invited the companies that we did not approach directly to participate in our paper-and-pencil survey. Moreover, we conducted surveys at recruitment talks and fairs for university students that involved manufacturing companies, we met the managers of R&D, manufacturing, marketing, human resource, and logistics departments. We treated the subsidiaries of group companies (e.g., CRRC Corporation Limited) as independent firms.

A total of 458 companies accepted our invitation to participate in the study. In the data cleaning process, we identified 86 respondents who were uninformed about the issues under investigation and 21 surveys with incomplete and unclear answers. Therefore, we obtained 351 questionnaires and a valid response rate of around 76.64%. The sample consisted of pharmaceutical and biotechnology (5.98%); textile, clothing, and furniture (3.13%); equipment manufacturing (23.08%); computer and communication (19.09%); automotive and transportation (23.93%); building and construction (13.11%); metal and mining (3.70%); and other firms (7.98%).

### Measurements

All the items (Table 1) were measured with a five-point Likert scale ranging from 1 (very weak) to 5 (very strong).

**Table 1:** Items, reliability, and validity.

Constructs	Items	Factor loading
**Stakeholder pressure** KMO = 0.754; AVE = 0.506; CR = 0.857; *α* = 0.866.	How important do you consider each of the following influences on your firm’s digitalization?	
	1. Government	0.615
	2. Shareholders	0.598
	3. Consumers/ Suppliers	0.838
	4. Employees	0.717
	5. Creditors	0.565
	6. Competitors	0.876
**Routine reconfiguration** KMO = 0.796; AVE = 0.519; CR = 0.811; *α* = 0.808.	1. Our firm will continuously adjust standard processes to achieve higher operational efficiency.	0.675
	2. Our firm proactively carries out organizational changes to meet new internal and external challenges.	0.803
	3. If our firm acquires new knowledge or technology, we will use it to improve process specifications.	0.720
	4. Our firm provides timely training and guidance on new organizational norms for employees.	0.675
**Strategic flexibility** KMO = 0.834; AVE = 0.574; CR = 0.889; *α* = 0.894.	How flexibly can your company react to different exogenous changes (e.g., shifts in economic conditions; and the emergence of an unexpected market opportunity)?	
	1. If circumstances change, our firm can easily change its current plans?	0.637
	2. If circumstances change, our firm is prepared to react in a modified and viable manner?	0.757
	3. If circumstances change, our firm can control a shift in strategy?	0.805
	4. If circumstances change, does our firm have the necessary practical knowledge to make shifts in daily routines and practices?	0.728
	5. If circumstances change, can our firm proactively develop a new project?	0.867
	6. If circumstances change, can our firm shift projects with a high probability of success?	0.733
**Digital process innovation** KMO = 0.788; AVE = 0.530; CR = 0.848; *α* = 0.809.	1. In the past three years, to what extent has your firm modified, and improved existing business processes via digital technologies?	0.783
	2. In the past three years, to what extent has your firm strengthened current digital technologies?	0.655
	3. In the past three years, to what extent has your firm invested in purchasing new digital technology for the process?	0.646
	4. In the past three years, to what extent has your firm created new business processes via digital technologies?	0.849
	5. In the past three years, to what extent has your firm invested in R&D on digitalization dedicated to process innovation?	0.686

#### 1. Stakeholder pressure

Consistent with [37, 43], we measured stakeholder pressure with six widely used items. The items can capture the main pressure faced by firms in industrialized and developing countries.

#### 2. Routine reconfiguration

According to previous studies, routine reconfiguration refers to variations in routines that are much more intentional and radical [51]. Hence, we employed the measurement of [63, 64] and observed routine reconfiguration by measuring routine changes and training for such changes.

#### 3. Strategic flexibility

Researchers constructed various items to mirror strategic flexibility. For instance, [21] addressed reactions to different exogenous changes, whereas other scholars highlighted firms’ flexibility of the resources and ability when encountering exogenous changes. As [65] posited, strategic flexibility depicts the proactive and reactive abilities of firms to react to environmental changes. Thus, we followed the approach of [21, 66] by specifying the exogenous changes and measuring the firms’ ability to deal with such changes.

#### 4. Digital process innovation

In line with [4], we measured digital process innovation by using five items that can indicate the significant changes induced by a firm’s introduction of digital technologies into its business process.

In addition, we controlled for firm age, firm size, and R&D intensity, as suggested by previous studies [4, 62]. We measured the firm age and size by calculating the number of years since a firm’s establishment and the number of employees, respectively. For R&D intensity, we used the share of R&D expenditures in total sales.

### Model setting and empirical strategies

By referring to the empirical research paradigm [28, 62], we developed five equations (models) for the hypothesis examination. The multi-regression method is a statistical analysis technique commonly used in innovation management to examine the degree to which two or more variables can explain another variable. This study involves direct, mediating, and moderating effects, and thus we created five equations.

To test the direct effect (i.e., H1), we created Eq (1). In Eq (1), the dependent variable is digital process innovation (DPI), the independent variable is stakeholder pressure (SP), and the control variables are represented by CVs. In addition, *α*_0_ is a constant, and ε is the random error of the model. If *α*_1_ is significantly positive, then the direct effect of stakeholder pressure on digital process innovation is supported, that is, H1 is confirmed.


$$y_{DPI}=\alpha_{0}+\alpha_{1}SP+\sum \alpha_{2}CVs+\varepsilon$$ (1)

To test the mediating effect (i.e., H2 and H3), we referred to [67, 68], created Eqs (2) and (3), and performed hierarchical regression analysis. In Eq (2), the dependent variable is routine reconfiguration (RR), and the independent variable is stakeholder pressure. If *β*_1_ is significantly positive, then H2 is verified. Furthermore, if the mediator functions γ_1_ and γ_2_ are significantly positive, and then, H3 is supported.


$$y_{RR}=\beta_{0}+\beta_{1}SP+\sum \beta_{2}CVs+\mu$$ (2)

$$y_{DPI}=\gamma_{0}+\gamma_{1}SP+\gamma_{2}RR+\sum \gamma_{3}CVs+\sigma$$ (3)

To test the moderating effect (i.e., H4a and H4b), we created Eqs (4) and (5). By referring to [69], we standardized the independent (SP) and moderating variables (SF) and constructed interaction terms (SPSF) to minimize the interference of multi-collinearity. If *δ*_3_ and *φ*_3_ are significantly positive, then H4a and H4b are verified.


$$y_{RR}={\delta \gamma }_{0}+\delta_{1}SP+\delta_{2}SF+\delta_{3}SPSF+\sum \delta_{4}CVs+\tau$$ (4)

$$y_{DPI}=\varphi_{0}+\varphi_{1}SP+\varphi_{2}SF+\varphi_{3}SPSF+\varphi_{4}RR+\sum \varphi_{5}CVs+\omega$$ (5)

The reliable findings were based on appropriate data analysis methods. By referring to the empirical research paradigm [28, 62], we comprehensively employed multiple methods, namely, reliability and validity analysis, descriptive statistical analysis, correlation analysis, common method bias testing, multi-collinearity testing, and multiple regression methods.

## Results of data analysis

### Reliability and validity

To check the reliability and validity of our latent constructs, we conducted a confirmatory factor analysis. The results showed that the comparative fit index was > 0.90, and the factor loading was > 0.5 (Table 1). Then, we calculated the composite reliability and average variance extracted (AVE), and the results revealed that the composite reliability of all the latent constructs was greater than 0.80 and the AVE values exceeded 0.50, which suggested adequate convergent validity and discriminant validity.

### Descriptive statistics and correlation

Table 2 presents the descriptive statistics and intercorrelations among the variables. As expected, stakeholder pressure, routine reconfiguration, and strategic flexibility were correlated with digital process innovation. Common method variance (CMV) may be a potential biasing threat that results in wrong conclusions about the relationships [28]. Therefore, we avoided CMV by conducting a pretest before formally conducting the survey and making revisions to the wording of the items. We also adopted Harman’s one-factor approach to examine the CMV. The results showed that the first factor accounted only for 18.87% of the total variance, which suggested that CMV did not bias our conclusions.

**Table 2:** Means, standard deviations, and correlations of the variables.

Variable	1	2	3	4	5	6	7
SP	-						
RR	0.485**	-					
SF	-0.081	0.034	-				
DPI	0.380**	0.303**	0.076	-			
Firm age	-0.006	-0.061	-0.021	-0.061	-		
Firm size	-0.060	0.063	-0.131*	-0.004	0.387**	-	
R&D intensity	0.090	0.059	0.002	0.174**	0.140**	0.178**	-
Mean	3.526	3.793	3.622	4.017	2.382	2.231	2.680
SD	0.426	0.476	0.637	0.452	1.048	0.944	0.783

** p<0.01

### Direct effect analysis and mediating effect analysis

To test H1, H2, and H3, we conducted ordinary least squares regression analysis in SPSS 25.0 (Table 3). As shown by the adjusted R^2^ value in Model 2, the main effect of the stakeholder pressure accounted for around 16.2% of the variance in digital process innovation (Model 2), and around 23.6% of the variance in routine reconfiguration (Model 6). Thus, stakeholder pressure had a significant and positive effect on digital process innovation (*β* = 0.391, *p* < 0.01) and routine reconfiguration (*β* = 0.548, *p* < 0.01). Hence, H1 and H2 were verified, respectively.

**Table 3:** Regression results.

Variable	DPI					RR	
	Model 1	Model 2	Model 3	Model 4	Model 5	Model 6	Model 7
SP		0.391** (0.052)	0.308** (0.059)	0.406** (0.052)	0.339** (0.060)	0.548** (0.053)	0.562** (0.052)
RR			0.151** (0.053)		0.120* (0.053)		
SF				0.078* (0.035)	0.070* (0.035)		0.065 (0.035)
Interaction term				0.062** (0.022)	0.054* (0.023)		0.068** (0.023)
Firm age	-0.037 (0.025)	-0.039 (0.023)	-0.041 (0.023)	-0.037 (0.023)	-0.039 (-0.023)	0.015 (0.023)	0.017 (0.023)
Firm size	-0.002 (0.028)	0.012 (0.026)	0.006 (0.026)	0.020 (0.026)	0.014 (0.026)	0.041 (0.026)	0.046 (0.026)
R&D intensity	0.108** (0.031)	0.086** (0.029)	0.086** (0.029)	0.075** (0.029)	0.077** (0.029)	-0.003 (0.029)	-0.014 (0.029)
Constant	3.821** (0.099)	2.475** (0.203)	2.212** (0.221)	2.152** (0.247)	1.975** (0.258)	1.743** (0.204)	1.474** (0.249)
*R* ^2^	0.038	0.171	0.190	0.201	0.212	0.245	0.271
Adjust *R*^2^	0.030	0.162	0.179	0.187	0.196	0.236	0.258
F	4.553**	17.891**	16.227**	14.409**	13.222**	28.015**	21.319**

* p<0.05

** p<0.01

Regression coefficients are unstandardized coefficients with standard error within parentheses.

To examine H3, we observed the coefficient of stakeholder pressure. The results of Model 3 showed that the coefficient of stakeholder pressure was 0.308 (*p* < 0.01), which was lower than the coefficient of stakeholder pressure in Model 2, and the coefficient of routine reconfiguration was positive and significant (*β* = 0.151, *p* < 0.01). To further prove the mediating effect, we used the PROCESS plug-in to estimate the mediating effect with the bootstrap method. We used the repeated sampling method (5,000 times) to construct bootstrap confidence intervals. The results revealed that the total effect of stakeholder pressure on digital process innovation was 0.391 (95%CI = 0.191,0.494), and the indirect effect of stakeholder pressure on digital process innovation was 0.083 (95%CI = 0.023, 0.162). Therefore, H3 was supported.

### Moderating effect analysis

To test H4a and H4b, we standardized the two predictors, namely, stakeholder pressure and strategic flexibility, before creating the interaction terms. The results of Model 7 showed that the interaction term was positive and significant (*β* = 0.068, *p* < 0.01). When added the mediator, the interaction term in Model 5 was also positive and significant (*β* = 0.054, *p* < 0.05), which indicated that H4a and H4b were supported. Furthermore, we employed the PROCESS plug-in to examine the moderated mediating effect. The results showed that the conditional direct and indirect effects under low (i.e., -1SD), medium, and high (i.e., +1SD) levels of strategic flexibility were statistically significant. The index of the moderated mediating was 0.030 [0.001,0.067], which further indicated that H4a and H4b were verified.

**Fig 2:**
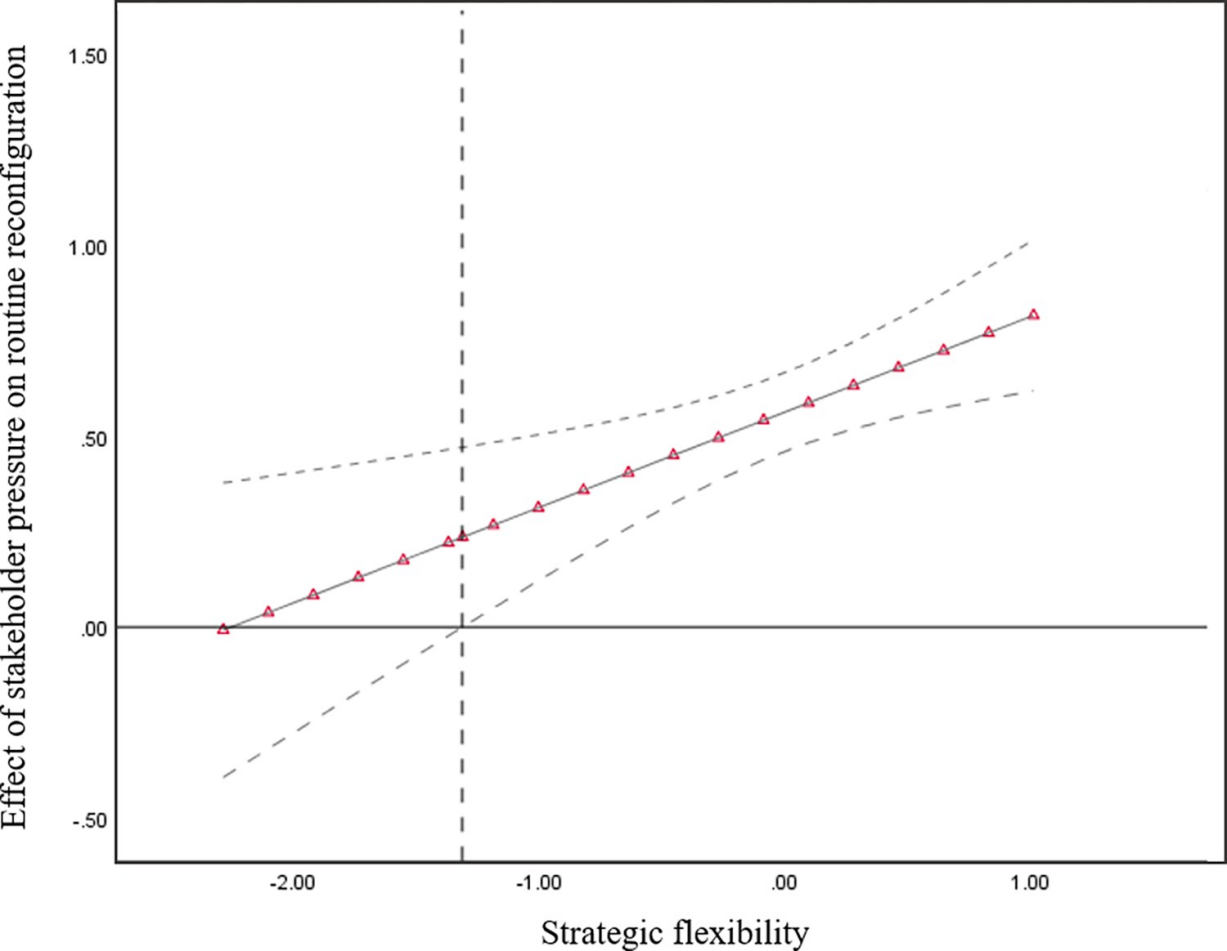
Moderating role of strategic flexibility on the relationship between stakeholder pressure and routine reconfiguration.

To interpret our findings effectively, we graphed the Johnson–Neyman plots. Fig 2 shows that when strategic flexibility is greater than -1.314, the positive relationship between stakeholder pressure and routine reconfiguration strengthened significantly; however, this was not the case for low levels.

**Fig 3:**
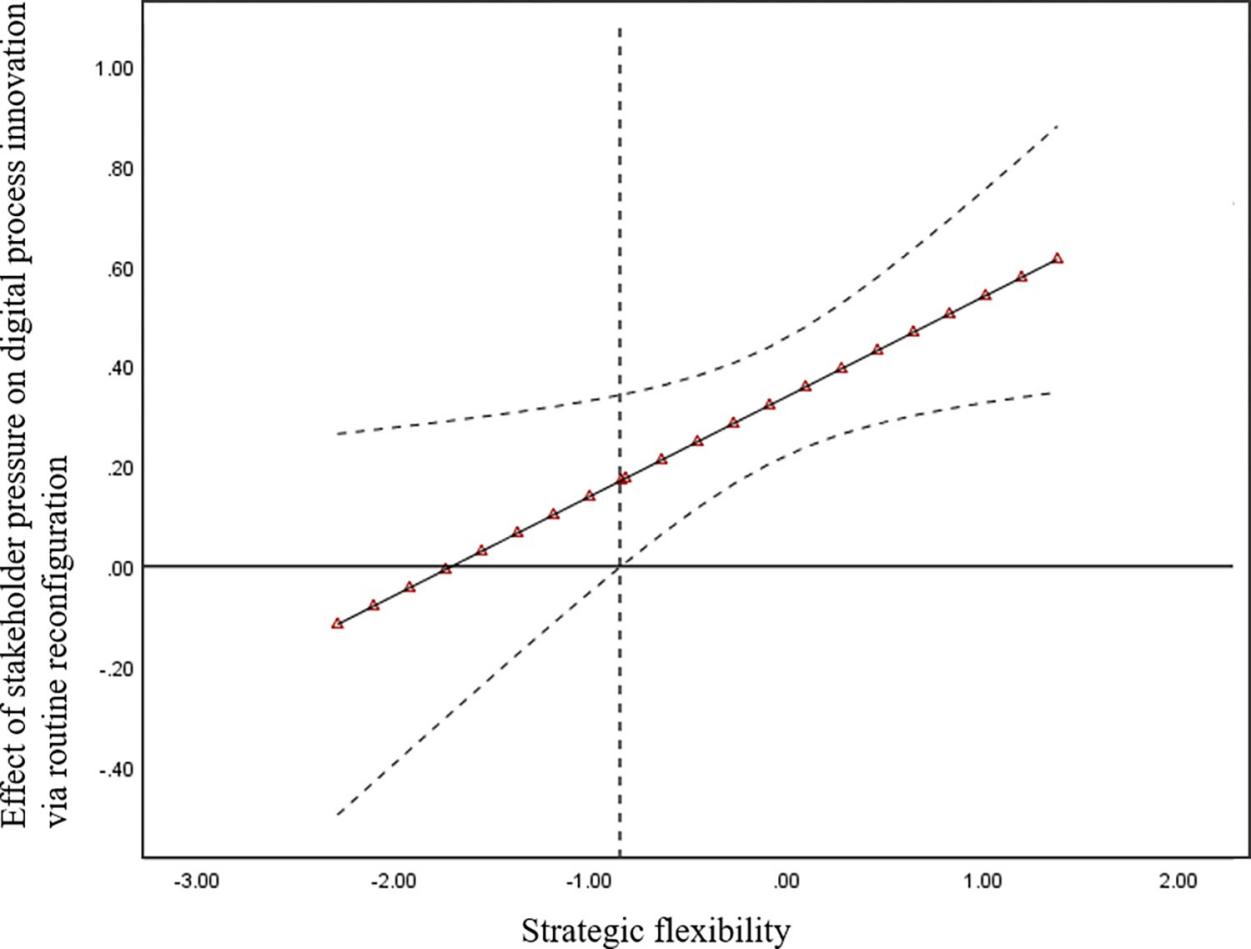
Moderating role of strategic flexibility on the relationship between stakeholder pressure and digital process innovation via routine reconfiguration.

Fig 3 illustrates that when strategic flexibility is greater than -0.844, the positive relationship between stakeholder pressure and digital process innovation via routine reconfiguration strengthened significantly.

## Conclusions and implications

### Conclusions

As mentioned previously, stakeholders paying growing attention to digitalization, which can be regarded as pressure on organizations. The prevalence of stakeholder pressure led researchers to investigate its role in reshaping digital process innovation in the digital age. We tested our hypotheses by using survey data collected from 351 Chinese firms.

The conclusions of this study are as follows: first, we observed the positive effect of stakeholder pressure on digital process innovation via routine reconfiguration. That is, perceiving the expectations and attention of stakeholders will push a firm to take a step toward reconfiguring its codified routines and developing new applicable routines to adapt to the digital era. Thus, our study is in line with previous stakeholder and digital innovation studies [4, 9, 20], which highlighted the significance of stakeholders in explaining organizational actions and decision-making.

Second, the mediating role of routine reconfiguration partially confirmed the findings of studies on routine dynamics, which highlighted the dynamic, variable, and emergent nature of a routine, while showing that the subsequent variation comes from either external or internal stimuli [52, 70]. Such practices can further prompt a firm to introduce and effectively absorb digital technologies into its processes to engage in digital process innovation.

Third, we demonstrated that, as the strategic flexibility of a firm increased, the positive effect of stakeholder pressure on its routine reconfiguration also increased. The finding is consistent with that of previous strategic flexibility studies [59, 60]. The introduction of digital technologies into established firms requires extra strategic attention from upper echelons and resources for new workflows, procedures, and routines. Such strategic changes and resource replenishment can enable the selection and application of an optimal routine, that is, the routine that will optimize the final routine reconfiguration.

## Discussion

The results of our study may be context-specific and thus should be viewed cautiously when generalized to other contexts. Digital technologies, such as AI and cloud computing, can help disrupt Chinese businesses. China unveiled its five-year plan to speed up the integration of digital and real economies amid its broader push to implement a policy framework for the nation’s industrial development until 2025. Thus, Chinese supply chains and the government have gradually made considerable investments in and set various policies for digitalization. Hence, pressure from the government and other stakeholders may be strong, as well as the infrastructure for creating digital process innovation. Although we have theoretical reasons to believe that firms in other countries that face strong stakeholder pressure may experience similar positive impacts, we were very tentative about generalizing our study to other settings. This speculation must be validated by future research in other economies, and the differences between China and other countries should be considered carefully.

### Theoretical implications

First, our study contributes to the digitalization and process innovation literature by extending process innovation to digital process innovation. Prior research observed the impact of process innovation on organizational outcomes and explored its determinants. Digital process innovation emerged with the increasing use of digital technology or its introduction into firms’ operating processes and systems; however, its importance has yet to be examined comprehensively [4, 71]. Digital process innovation involves not only digitizing the undigitized processes through the use of digital technologies, digital devices, digital infrastructure, and data but also the domains that have been digitized [33]. Thus, digital process innovation is a means for changing and facilitating new pathways of action that may produce dramatic side effects [33]. In line with the call to develop a convergent logic for digitalization and process innovation, our study extended process innovation and addressed the established concept of digital process innovation.

Second, our study offers new insights into the development of digital process innovation by highlighting the impact of stakeholder pressure on digitalization and routine reconfiguration. To the best of our knowledge, the literature on digital process innovation is limited. Scholars called for the development of a multi-stakeholder perspective to examine digitalization issues owing to the multiple stakeholders involved in digitalization [16]. According to [41, 72], stakeholders play an influential role in a firm’s growth, and stakeholder pressure can result in significant motivation for certain organizational actions. Against this backdrop, we employed stakeholder theory to investigate corporations’ underlying digital process innovation tactics. Specifically, we proposed and proved that stakeholder pressure for digitalization enabled firms to engage in digital process innovation. Moreover, to effectively understand the micro-foundations of this causal relationship, we posited and demonstrated that the relationship was mediated by a firm’s ability to reconfigure its routines and implementations. Thus, we contribute to ongoing efforts to provide an empirically examined digital process innovation conceptual framework.

Third, our study contributes to stakeholder theory by highlighting the impact of strategic flexibility. At the general level, our findings share similarities with those of previous stakeholder studies that emphasized that response to stakeholders’ concerns can induce organizational policies and changes (e.g., firms’ innovation investment and outcomes; [13, 43, 73]. However, our study partially challenges previous studies. Not all firms can extract the highest gains from stakeholder pressure, because the strategic flexibilities of firms vary. If a firm has low competence to switch from old strategic actions and resources that can hardly be redeployed, then the pressure exerted by stakeholders may not be as effective as expected. To a certain extent, our study proved the theory of [74], which indicated that considerable slack resources in a firm’s responsiveness to stakeholder pressure will be highly effective. Thus, our study provides new insights into the context and mechanism t through which stakeholder pressure affects firms’ digital process innovation.

### Managerial implications

Our study has implications for practitioners of firms. Deploying digital process innovation can not only boost productivity and efficiency but also contribute to improved health and safety conditions in the workplace as well as in society, which can lead to a competitive advantage. However, digital process innovation takes time, costs money, and requires substantial dedicated resources [7]. Thus, how digital process innovation can be developed has become a challenge for practitioners. Our findings present a window of opportunity, that is, stakeholder pressure for digitalization can encourage a firm to step up in developing its digital process innovation. Addressing the interests of multiple stakeholders is a form of “enlightened self-interest” and the intrinsic value of a firm [38]. Reactively or proactively responding to stakeholder pressure can help firms introduce digital technologies and make processual changes. Moreover, stakeholder pressure is typically accompanied by specialized and advanced knowledge from stakeholders that can facilitate a firm’s digital process application. Therefore, managers must cultivate their ability to transform stakeholder pressure into motivation, take full use of external environmental pressure to change existing organizational practices, and promote innovation in digital processes.

In addition, when dealing with digitalization pressure from stakeholders, managers can use a repertoire of flexible strategies as an aid to supplement and strengthen the firm’s digitalization in a timely manner. With the increasing irregularity, complexity, uncertainty, and dynamism of most markets and competitive environments, strategic flexibility has become fundamental to organizational adaptation [55]. Specifically, a flexible strategy can offer proactive, resilient, and open-minded insights that are important for exploiting innovation opportunities under stakeholder pressure and allocating existing resources to realize them. Thus, firms should intentionally maintain a flexible strategic technique and build, and improve their flexibility to integrate and deploy resources.

### Social implications

Our study also has social implications. The government, as a strong stakeholder, has substantial control over critical resources and can shape the operating environment of a corporation [8]. Our findings demonstrated that the pressure exerted by the government on firms can give rise to digital process innovation. Thus, policymakers should provide digital initiatives (tax incentives, financial support, or digital projects) to shape firms’ attention allocation and induce digital process innovation.

### Limitations and future research

This study has some limitations that can offer valuable directions for future research. First, this study employed a cross-sectional questionnaire to test the theoretical hypotheses, which was an inherent downside of this research. According to [75], cross-sectional data cannot describe dynamic changes in constructs. That is, the varying levels of pressure from stakeholders and digital process innovation cannot be observed by the survey data. Hence, future longitudinal studies should investigate the extent to which the measurement reflects changes in stakeholders’ pressure and digital process innovation over time.

Second, with the continuous green pressure on resources and the environment [76], future research can consider the important role of digital technologies in industrial structure upgrading, especially in green industries (e.g., the construction industry).

Third, along with the degree of digitalization, the importance of stakeholders in boosting digital process innovation may vary. The interests of stakeholders may also be divergent. Conflicts among stakeholders have yet to be examined thoroughly and require further research.

Last, future competition in the digital era will be the competition among digital ecosystems that can increase the interactions among stakeholders. Thus, future studies can consider the development and features of the digital ecosystem to explain one action to engage in digital process innovation.

## References

[1] Sorescu A, Schreier M. Innovation in the digital economy: a broader view of its scope, antecedents, and consequences. J Acad Market Sci. 2021;49(4):627–31. 10.1007/s11747-021-00793-z.

[2] Kamalaldin A, Sjödin D, Hullova D, Parida V. Configuring ecosystem strategies for digitally enabled process innovation: A framework for equipment suppliers in the process industries. Technovation. 2021;105:102250. 10.1016/j.technovation.2021.102250.

[3] Kohtamäki M, Parida V, Oghazi P, Gebauer H, Baines T. Digital servitization business models in ecosystems: a theory of the firm. J Bus Res. 2019;104:380–92. 10.1016/j.jbusres.2019.06.027.

[4] Zhao Y, Kong S. Firms’ openness in specialized search and digital innovation among process-oriented mining enterprises: a moderated mediation model. Resources Policy. 2022;75:102466. 10.1016/j.resourpol.2021.102466.

[5] Nwankpa JK, Roumani Y, Datta P. Process innovation in the digital age of business: the role of digital business intensity and knowledge management. J Knowl Manag. 2022;26(5):1319–41. 10.1108/JKM-04-2021-0277.

[6] Sjödin DR, Parida V, Leksell M, Petrovic A. Smart factory implementation and process innovation. Res Technol Manage. 2018;61(5):22–31. 10.1080/08956308.2018.1471277.

[7] Chirumalla K. Building digitally-enabled process innovation in the process industries: a dynamic capabilities approach. Technovation. 2021;105:102256. 10.1016/j.technovation.2021.102256.

[8] Wang X, Li Y, Tian L, Hou Y. Government digital initiatives and firm digital innovation: evidence from China. Technovation. 2023:102545. 10.1016/j.technovation.2022.102545.

[9] Yin S, Zhao Y. Digital green value co-creation behavior, digital green network embedding and digital green innovation performance: moderating effects of digital green network fragmentation. Hum Soc Sci Commun. 2024;11(1):228. 10.1057/s41599-024-02691-5.

[10] Svahn F, Mathiassen L, Lindgren R. Embracing digital innovation in incumbent firms: how Volvo cars managed competing concerns. MIS Quart. 2017;41:239–53. 10.25300/MISQ/2017/41.1.12.

[11] Freeman RE. Strategic management: A stakeholder approach. Boston: Pitman; 1984.

[12] Marcon Nora GA, Alberton A, Ayala DHF. Stakeholder theory and actor-network theory: the stakeholder engagement in energy transitions. Bus Strateg Environ. 2023;32(1):673–85. 10.1002/bse.3168.

[13] Shi Y, Tsai K-H. A sequential process from external stakeholder pressures to performance in services. J Serv Theor Pract. 2022;32(5):589–619. 10.1108/JSTP-06-2021-0109.

[14] Cantor D E., Blackhurst J, Pan M, Crum M. Examining the role of stakeholder pressure and knowledge management on supply chain risk and demand responsiveness. Int J Logist Manag. 2014;25(1):202–23. https://doi.org/ 10.1108/IJLM-10-2012-0111.

[15] Dmytriyev SD, Freeman RE, Hörisch J. The relationship between stakeholder theory and corporate social responsibility: differences, similarities, and implications for social issues in management. J Manage Stud. 2021;58(6):1441–70. 10.1111/joms.12684.

[16] Broekhuizen TLJ, Broekhuis M, Gijsenberg MJ, Wieringa JE. Introduction to the special issue–Digital business models: a multi-disciplinary and multi-stakeholder perspective. J Bus Res. 2021;122:847–52. 10.1016/j.jbusres.2020.04.014.

[17] Abrell T, Pihlajamaa M, Kanto L, vom Brocke J, Uebernickel F. The role of users and customers in digital innovation: insights from B2B manufacturing firms. Inform Manage-Amster. 2016;53(3):324–35. 10.1016/j.im.2015.12.005.

[18] Bridoux F, Stoelhorst JW. Microfoundations for stakeholder theory: managing stakeholders with heterogeneous motives. Strategic Manage J. 2014;35(1):107–25. 10.1002/smj.2089.

[19] Garcés‐Ayerbe C, Rivera‐Torres P, Murillo‐Luna JL. Stakeholder pressure and environmental proactivity. Manage Decis. 2012;50(2):189–206. https://doi.org/ 10.1108/00251741211203524.

[20] Yin S, Zhang N, Ullah K, Gao S. Enhancing digital innovation for the sustainable transformation of manufacturing industry: a pressure-state-response system framework to perceptions of digital green innovation and its performance for green and intelligent manufacturing. Systems-Basel. 2022;10(3):72. 10.3390/systems10030072.

[21] Hensellek S, Kleine-Stegemann L, Kollmann T. Entrepreneurial leadership, strategic flexibility, and venture performance: does founders’ span of control matter? J Bus Res. 2023;157:113544. 10.1016/j.jbusres.2022.113544.

[22] Zang S, Wang H, Zhou J. Impact of eco-embeddedness and strategic flexibility on innovation performance of non-core firms: the perspective of ecological legitimacy. J Innov Knowl. 2022;7(4):100266. 10.1016/j.jik.2022.100266.

[23] Utterback JM, Abernathy WJ. A dynamic model of process and product innovation. Omega-Int J Manage S. 1975;3(6):639–56. 10.1016/0305-0483(75)90068-7.

[24] Brem A, Nylund PA, Schuster G. Innovation and de facto standardization: the influence of dominant design on innovative performance, radical innovation, and process innovation. Technovation. 2016;50–51:79–88. 10.1016/j.technovation.2015.11.002.

[25] Wang L, Jin JL, Zhou KZ. Technological capability strength/asymmetry and supply chain process innovation: the contingent roles of institutional environments. Research Policy. 2023;52(4):104724. 10.1016/j.respol.2023.104724.

[26] Aliasghar O, Rose EL, Asakawa K. Sources of knowledge and process innovation: the moderating role of perceived competitive intensity. Int Bus Rev. 2022;31(2):101920. 10.1016/j.ibusrev.2021.101920.

[27] Najafi-Tavani S, Najafi-Tavani Z, Naudé P, Oghazi P, Zeynaloo E. How collaborative innovation networks affect new product performance: product innovation capability, process innovation capability, and absorptive capacity. Ind Market Manag. 2018;73:193–205. 10.1016/j.indmarman.2018.02.009.

[28] Cherrafi A, Garza-Reyes JA, Kumar V, Mishra N, Ghobadian A, Elfezazi S. Lean, green practices and process innovation: a model for green supply chain performance. Int J Prod Econ. 2018;206:79–92. 10.1016/j.ijpe.2018.09.031.

[29] Xie X, Hoang TT, Zhu Q. Green process innovation and financial performance: the role of green social capital and customers’ tacit green needs. J Innov Knowl. 2022;7(1):100165. 10.1016/j.jik.2022.100165.

[30] Lefebvre L-A, Lefebvre E, Colin D. Process innovation, productivity, and competitiveness in smaller manufacturing firms. Can J Adm Sci. 1991;8(1):19–28. 10.1111/j.1936-4490.1991.tb00659.x.

[31] Reichstein T, Salter A. Investigating the sources of process innovation among UK manufacturing firms. Ind Corp Change. 2006;15(4):653–82. 10.1093/icc/dtl014.

[32] Martínez-Alonso R, Martínez-Romero MJ, Rojo-Ramírez AA, Lazzarotti V, Sciascia S. Process innovation in family firms: family involvement in management, R&D collaboration with suppliers, and technology protection. J Bus Res. 2023;157:113581. 10.1016/j.jbusres.2022.113581.

[33] Mendling J, Pentland BT, Recker J. Building a complementary agenda for business process management and digital innovation. Eur J Inform Syst. 2020;29(3):208–19. 10.1080/0960085X.2020.1755207.

[34] Liu Y, Dong JY, Wei J. Digital Innovation Management: theoretical Framework and Future Research. J Manag World. 2020;36(07):198–217+9. 10.19744/j.cnki.11-1235/f.2020.0111.

[35] Richard J. Boland J, Lyytinen K, Yoo Y. Wakes of innovation in project networks: the case of digital 3D representations in architecture, engineering, and construction. Organ Sci. 2007;18(4):631–47. 10.1287/orsc.1070.0304.

[36] Bunduchi R, Crișan-Mitra C, Salanță II, Crișan EL. Digital product innovation approaches in entrepreneurial firms–the role of entrepreneurs’ cognitive frames. Technol Forecast Soc. 2022;175:121343. 10.1016/j.techfore.2021.121343.

[37] Shubham, Charan P, Murty LS. Secondary stakeholder pressures and organizational adoption of sustainable operations practices: the mediating role of primary stakeholders. Bus Strateg Environ. 2018;27(7):910–23. 10.1002/bse.2041.

[38] de Gooyert V, Rouwette E, van Kranenburg H, Freeman E. Reviewing the role of stakeholders in operational research: a stakeholder theory perspective. Eur J Oper Res. 2017;262(2):402–10. 10.1016/j.ejor.2017.03.079.

[39] Donaldson T, Preston LE. The Stakeholder Theory of the corporation: concepts, evidence, and implications. Acad Manage Rev. 1995;20(1):65–91. 10.2307/258887.

[40] Egels-Zandén N, Sandberg J. Distinctions in descriptive and instrumental stakeholder theory: a challenge for empirical research. Bus Ethics. 2010;19(1):35–49. 10.1111/j.1467-8608.2009.01577.x.

[41] Rubio-Andrés M, Ramos-González MdM, Sastre-Castillo MÁ, Gutiérrez-Broncano S. Stakeholder pressure and innovation capacity of SMEs in the COVID-19 pandemic: mediating and multigroup analysis. Technol Forecast Soc. 2023;190:122432. doi: 10.1016/j.techfore.2023.122432 36816868 PMC9928774

[42] Singh SK, Del Giudice M, Chiappetta Jabbour CJ, Latan H, Sohal AS. Stakeholder pressure, green innovation, and performance in small and medium-sized enterprises: the role of green dynamic capabilities. Bus Strateg Environ. 2022;31(1):500–14. 10.1002/bse.2906.

[43] Kawai N, Strange R, Zucchella A. Stakeholder pressures, EMS implementation, and green innovation in MNC overseas subsidiaries. Int Bus Rev. 2018;27(5):933–46. 10.1016/j.ibusrev.2018.02.004.

[44] Liang L, Li Y. How does government support promote digital economy development in China? The mediating role of regional innovation ecosystem resilience. Technol Forecast Soc. 2023;188:122328. 10.1016/j.techfore.2023.122328.

[45] Andrew Petersen J, Paulich BJ, Khodakarami F, Spyropoulou S, Kumar V. Customer-based execution strategy in a global digital economy. Int J Res Mark. 2022;39(2):566–82. 10.1016/j.ijresmar.2021.09.010.

[46] Cavallo A, Ghezzi A, Dell’Era C, Pellizzoni E. Fostering digital entrepreneurship from startup to scaleup: the role of venture capital funds and angel groups. Technol Forecast Soc. 2019;145:24–35. 10.1016/j.techfore.2019.04.022.

[47] Wimelius H, Sandberg J, Olsson M, Gunhaga M. Navigating the volatile world of digital entrepreneurship. Bus Horizons. 2023. 10.1016/j.bushor.2023.05.001.

[48] Nelson R, Winter S. An Evolutionary Theory of Economic Change. Bibliovault OAI Repository, the University of Chicago Press. 1982;32. 10.2307/2393143.

[49] Davies A, Frederiksen L, Cacciatori E, Hartmann A. The long and winding road: routine creation and replication in multi-site organizations. Research Policy. 2018;47(8):1403–17. 10.1016/j.respol.2018.04.016.

[50] Chen JE, Pan SL, Ouyang TH. Routine reconfiguration in traditional companies’ e-commerce strategy implementation: a trajectory perspective. Inform Manage-Amster. 2014;51(2):270–82. 10.1016/j.im.2013.11.008.

[51] Chen J, Guo X, Zhao H. Cross-fertilization for routine reconfiguration in IT-enabled organizational transformation. Inform Manage-Amster. 2021;58(2):103414. 10.1016/j.im.2020.103414.

[52] Riviere M, Bass AE, Andersson U. Dynamic capability development in multinational enterprises: reconciling routine reconfiguration between the headquarters and subsidiaries. Glob Strateg J. 2021;11(3):380–401. 10.1002/gsj.1389.

[53] Bettinazzi ELM, Zollo M. Stakeholder orientation and experiential learning: evidence from corporate acquisitions. J Manage Stud. 2022;59(6):1422–59. 10.1111/joms.12782.

[54] Kohli R, Melville NP. Digital innovation: a review and synthesis. Information Systems Journal. 2019;29(1):200–23. 10.1111/isj.12193.

[55] Herhausen D, Morgan RE, Brozović D, Volberda HW. Re-examining strategic flexibility: a meta-analysis of its antecedents, consequences and contingencies. Brit J Manage. 2021;32(2):435–55. 10.1111/1467-8551.12413.

[56] Chan ATL, Ngai EWT, Moon KKL. The effects of strategic and manufacturing flexibilities and supply chain agility on firm performance in the fashion industry. Eur J Oper Res. 2017;259(2):486–99. 10.1016/j.ejor.2016.11.006.

[57] Sanchez R. Preparing for an Uncertain Future. Int Stud Manag Org. 1997;27(2):71–94. 10.1080/00208825.1997.11656708.

[58] Sanchez R. Strategic flexibility in product competition. Strategic Manage J. 1995;16(S1):135–59. 10.1002/smj.4250160921.

[59] Li Y, Su Z, Liu Y. Can strategic flexibility help firms profit from product innovation? Technovation. 2010;30(5):300–9. 10.1016/j.technovation.2009.07.007.

[60] Han C, Zhang S. Multiple strategic orientations and strategic flexibility in product innovation. Eur Res Manag Bus Ec. 2021;27(1):100136. 10.1016/j.iedeen.2020.100136.

[61] Björkdahl J. Strategies for digitalization in manufacturing firms. Calif Manage Rev. 2020;62(4):17–36. 10.1177/0008125620920349.

[62] Pesch R, Endres H, Bouncken RB. Digital product innovation management: balancing stability and fluidity through formalization. J Prod Innovat Manag. 2021;38(6):726–44. 10.1111/jpim.12609.

[63] Wang YW, Ma J, Wu XF, Liu SC. A study on the relationship between the behavior of the transformational leadership, the orientation to organizational learning, and the updating of organizational routines. Journal of Management World. 2012;(09):110–9. 10.19744/j.cnki.11-1235/f.2012.09.011.

[64] Gao Y, Ge B, Jiang D. A study about the relationships between organizational learning,organizational routines renewing and competitive advantage-under different levels of environmental uncertainty. Stud Sci Sci. 2017;35(09):1386–95. 10.16192/j.cnki.1003-2053.2017.09.013.

[65] Brozovic D. Strategic flexibility: a review of the literature. Int J Manag Rev. 2018;20(1):3–31. 10.1111/ijmr.12111.

[66] Miroshnychenko I, Strobl A, Matzler K, De Massis A. Absorptive capacity, strategic flexibility, and business model innovation: empirical evidence from Italian SMEs. J Bus Res. 2021;130:670–82. 10.1016/j.jbusres.2020.02.015.

[67] Baron RM, Kenny DA. The moderator–mediator variable distinction in social psychological research: conceptual, strategic, and statistical considerations. J Pers Soc Psychol. 1986;51:1173–82. doi: 10.1037//0022-3514.51.6.1173 3806354

[68] Wen ZL, Ye J. Analyses of Mediating Effects: The development of methods and models. Adv Psych Sci.2014;22(5):731–45. https://doi.org/CNKI:SUN:XLXD.0.2014-05-001.

[69] Aiken LS, West SG. Multiple regression: testing and interpreting interactions. Thousand Oaks, CA, US: Sage Publications, Inc; 1991.

[70] Turner SF, Rindova VP. Watching the clock: action timing, patterning, and routine performance. Acad Manage J. 2018;61(4):1253–80. 10.5465/amj.2015.0947.

[71] Laubengaier DA, Cagliano R, Canterino F. It takes two to tango: analyzing the relationship between technological and administrative process innovations in Industry 4.0. Technol Forecast Soc. 2022;180:121675. 10.1016/j.techfore.2022.121675.

[72] Sarkis J, Gonzalez-Torre P, Adenso-Diaz B. Stakeholder pressure and the adoption of environmental practices: the mediating effect of training. J Oper Manag. 2010;28(2):163–76. 10.1016/j.jom.2009.10.001.

[73] Yunus S, Elijido-Ten EO, Abhayawansa S. Impact of stakeholder pressure on the adoption of carbon management strategies. Sustain Account Mana. 2020;11(7):1189–212. 10.1108/SAMPJ-04-2019-0135.

[74] Xiao C, Wang Q, van Donk DP, van der Vaart T. When are stakeholder pressures effective? An extension of slack resources theory. Int J Prod Econ. 2018;199:138–49. 10.1016/j.ijpe.2018.03.002.

[75] Jin Y, Shao YF. Power-leveraging paradox and firm innovation: the influence of network power, knowledge integration and breakthrough innovation. Ind Market Manag. 2022;102:205–15. 10.1016/j.indmarman.2022.01.007.

[76] Dong T, Yin S, Zhang N. New energy-driven construction industry: digital green innovation investment project selection of photovoltaic building materials enterprises using an integrated fuzzy decision approach. Systems-Basel. 2023;11(1):11. 10.3390/systems11010011.

